# p62/SQSTM1 Condensation Modulates Mitochondrial Clustering to Participate in Mitochondrial Quality Control

**DOI:** 10.1111/acel.70402

**Published:** 2026-02-11

**Authors:** Shan Sun, Jiaqi Xin, Yingping Zhang, Bojun Yang, Dan Su, Rui Ni, Qilian Ma, Ningning Li, Guoqiang Ma, Qiang Peng, Siqian Chen, Jochen H. M. Prehn, Kin Yip Tam, Hongfeng Wang, Zheng Ying

**Affiliations:** ^1^ Jiangsu Key Laboratory of Drug Discovery and Translational Research for Brain Diseases, College of Pharmaceutical Sciences Suzhou Medical College of Soochow University Suzhou Jiangsu China; ^2^ Faculty of Health Sciences University of Macau Macau China; ^3^ Department of Physiology & Medical Physics and FUTURE‐NEURO Research Centre Royal College of Surgeons in Ireland Dublin 2 Ireland; ^4^ MOE Key Laboratory of Geriatric Diseases and Immunology, College of Pharmaceutical Sciences Suzhou Medical College of Soochow University Suzhou Jiangsu China; ^5^ Jiangsu Province Engineering Research Center of Precision Diagnostics and Therapeutics Development Soochow University Suzhou Jiangsu China

## Abstract

Mitochondrial quality control is tightly associated with aging‐related neurodegenerative diseases such as Parkinson's disease, Alzheimer's disease, amyotrophic lateral sclerosis (ALS), and frontotemporal dementia (FTD). Previous studies reported that ALS/FTD‐associated protein p62 drives “mitochondrial clustering” (perinuclear clustering of fragmented and swollen mitochondria) during PINK1/Parkin‐mediated mitophagy, but the underlying molecular mechanism, especially the precise role of p62 in mitochondrial clustering during mitophagy and the potential relationship between the mitochondrial quality control mediated by p62 and disease pathogenesis of ALS/FTD, remains unclear. Here, using cell biology in combination with an optogenetic tool, we show that the phase separation (condensation) of p62 mediates the clustering of damaged mitochondria to form “grape‐like” clusters during PINK1/Parkin‐mediated mitophagy, which is tightly associated with aging‐related neurodegenerative diseases. In addition, our data suggest this mitochondrial clustering process is an arrest mechanism driven by p62 condensation (beyond the function of other autophagy receptors in mitophagy), which acts as a “brake” to reduce the surface area of dysfunctional mitochondria within cytoplasm for minimizing mitochondrial turnover in cells. Moreover, ALS/FTD‐related pathological mutations perturb p62 condensation, thereby inhibiting mitochondrial clustering and destroying the “brake” machinery of mitochondrial quality control. Together, our data highlight how p62 condensation modulates organelle quality control in cell biology, and the important role of p62 condensation in both physiology and pathology.

## Graphical abstract

In normal PINK1/Parkin‐mediated mitophagy, unlike OPTN (the mitophagy receptor that mediates ATG8‐labeled autophagosome recruitment onto ubiquitinated mitochondria), p62 mediates the clustering of ubiquitinated mitochondria to form the “grape‐like” aggregates with ubiquitin. Under aging‐related neurodegenerative disease conditions, pathogenic mutants perturb p62 condensation, thereby destroying mitochondrial aggregation and impairing mitochondrial quality control.

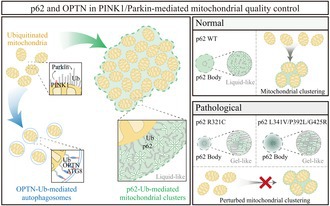

## 1 Introduction

Mitochondrial quality control is closely linked to aging‐related neurodegenerative disease proteins including Parkinson's disease (PD)‐linked PINK1/Parkin, Alzheimer's disease (AD)‐linked amyloid‐β and tau, amyotrophic lateral sclerosis (ALS), and frontotemporal dementia (FTD)‐linked TBK1/OPTN and UBQLN2 (Pickrell and Youle 2015; Ling et al. 2013; Palmer et al. 2025; Fang et al. 2019). Mitophagy is one form of selective autophagy that eliminates damaged or dysfunctional mitochondria to ensure intracellular mitochondrial quality control (Pickles et al. 2018; Narendra et al. 2008). In PINK1/Parkin‐mediated mitophagy, autophagosomes selectively recognize highly ubiquitinated mitochondria via linkages of autophagy receptors (OPTN and NDP52) for lysosomal degradation (Pickles et al. 2018; Lazarou et al. 2015). Mitophagy plays an important role in the quality control of mitochondria in aging‐related neurodegenerative diseases, and replenishment of nicotinamide adenine dinucleotide to modulate mitophagy showed neuroprotective effects on the treatment of PD, AD, and ALS in recent clinical trials (Palmer et al. 2025; Fang et al. 2019; Lautrup et al. 2019; Brakedal et al. 2022). Most studies on autophagy receptors, including p62/SQSTM1 (hereinafter referred to as p62), OPTN, NDP52, and others, have focused on the “receptor” role in autophagic recognition, characterizing how those receptors drive the association between cargos and ATG8s (LC3A, LC3B, LC3C, GABARAP, GABARAPL1, and GABARAPL2)‐labeled autophagosomes. However, recent studies showed that OPTN and NDP52 also regulate mitophagy initiation, suggesting multiple roles of these traditional receptors in the regulation of autophagy (Vargas et al. 2019; Nguyen et al. 2023; Ravenhill et al. 2019). Notably, as a classic autophagy receptor and an ALS/FTD‐associated protein, p62 is not always essential for autophagosome recruitment or mitochondrial degradation (Wong and Holzbaur 2014; Poon et al. 2021), but is critical for perinuclear “grape‐like” mitochondrial clustering during mitophagy (Okatsu et al. 2010; Narendra et al. 2010; Hsieh and Yang 2019). In this study, we aim to study the underlying molecular mechanism and the biological role of p62‐mediated mitochondrial clustering in PINK1/Parkin‐mediated mitophagy.

Intracellular p62 can form cytoplasmic p62 bodies through phase separation (Bjørkøy et al. 2005; Sun et al. 2018), the process in which biomolecules spontaneously and reversibly de‐mix from solution to form separated condensates (Shin and Brangwynne 2017). As the liquid droplets, the p62 bodies undergo droplet fusion and molecular exchange with the surrounding cytosolic components (Sun et al. 2018). Structurally, p62 consists of Phox and Bem1p (PB1), ZZ‐type zinc finger (ZZ), LC3‐interacting region (LIR), and ubiquitin‐associating (UBA) domains, and the formation of p62 bodies requires N‐terminal PB1 domain‐driven oligomerization and the multivalent interaction between the C‐terminal UBA domain of p62 and poly‐ubiquitin (poly‐Ub) chains as the driving force (Sun et al. 2018). Recent studies showed that p62 condensation regulates protein quality control including protein aggregation and degradation (Kurusu et al. 2023; Gallagher and Holzbaur 2023). However, it is unclear whether p62 condensation contributes to the quality control of membrane‐bound organelles in cells, and whether it is disrupted in ALS or FTD disease pathogenesis.

Here, we report that phase‐separated p62 acts as a driver for “grape‐like” cluster formation of ubiquitinated mitochondria during PINK1/Parkin‐mediated mitophagy, and ALS/FTD‐associated mutations perturb p62 phase separation, thereby disrupting p62‐mediated mitochondrial clustering. Our study deciphers the role of phase‐separated p62 in the “clustering” of damaged mitochondria, suggesting an unrecognized p62 function in the regulation of mitophagy in parallel with other autophagy receptors such as OPTN.

## 2 Results

### 2.1 Liquid‐Like Feature of p62 Bodies Is Impaired by ALS/FTD‐Linked Mutations

Endogenous and exogenous p62 have been reported to form droplet‐like bodies by phase separation in cultured cells (Bjørkøy et al. 2005; Sun et al. 2018). We observed the fast fusion and fission process between p62 bodies in cells and in vitro (Figure S1A,B) and fluorescence recovery after photobleaching (FRAP; Figure S1C,D), indicating the liquid‐like feature of p62 bodies in live HEK 293 cells, similar to the previous study reported (Sun et al. 2018).

**FIGURE 1:**
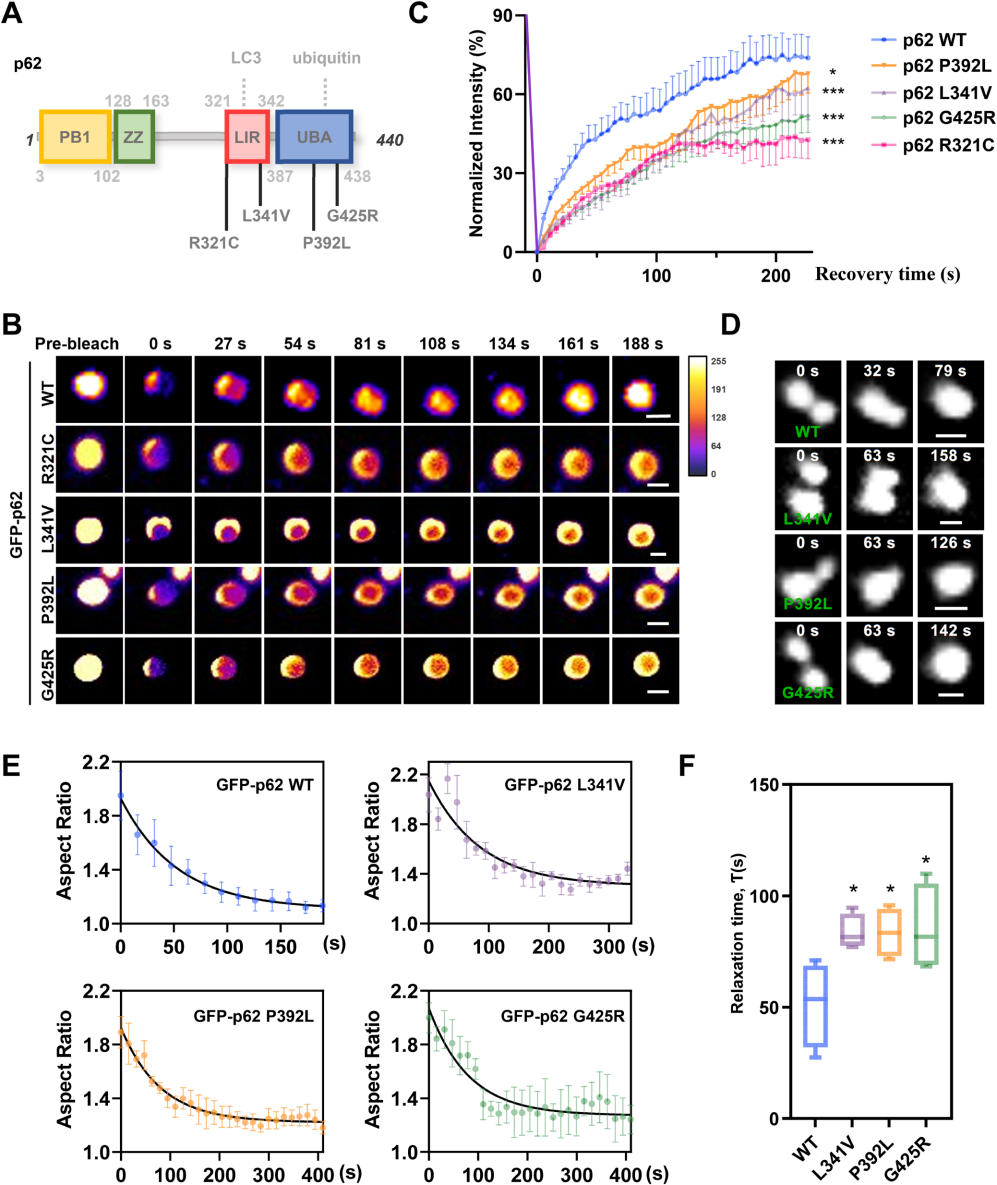
ALS/FTD‐associated mutations impede the liquidity of p62 phase separation. (A) Schematic diagram of p62 constructs and ALS/FTD‐associated mutations. Phox and Bem1p, PB1; ZZ‐type zinc finger, ZZ; LC3‐interacting region, LIR; ubiquitin‐associating, UBA. (B) HEK 293 cells were transfected with GFP‐p62 WT, R321C, L341V, P392L, or G425R for 24 h, and then representative images of FRAP analysis about these droplets were shown. Scale bar, 1 μm. (C) Quantification of fluorescence intensity of GFP‐p62 puncta in (B). Quantification data are shown as mean ± SEM from three independent experiments. *p < 0.05, ***p < 0.001, one‐way Anova followed by post hoc Tukey's tests. (D) HEK 293 cells were transfected with GFP‐p62 WT, L341V, P392L, or G425R for 24 h, and then representative time series of droplet fusion at the indicated time points were shown. Scale bar, 1 μm. (E, F) Aspect ratio as a function of time was fit to an exponential equation of decay (E) to determine *τ* (F). Boxplot (min‐max) of relaxation time *τ* for experiments in (D). *p < 0.05, one‐way Anova followed by post hoc Tukey's tests.

Several ALS/FTD‐associated p62 missense mutations (absent in the control population) have been reported (Fecto et al. 2011; Chen et al. 2014; Rea et al. 2014), but it is still unclear whether these mutations influence phase separation of p62. To investigate this, we generated a series of p62 mutants, including R321C, L341V, P392L, and G425R (Figure 1A; Fecto et al. 2011; Teyssou et al. 2013), and analyzed their phase separation behavior. The results showed that p62 mutations exhibited slower FRAP and a more immobile fraction than wildtype (WT), indicating these p62 mutations impair the dynamics of p62 bodies (Figure 1B,C, Figure S1E). Moreover, we used a droplet fusion assay to determine the effect of these mutations on p62 condensate dynamics and liquidity (Figure 1D), and the statistical results about fusion timescale (as indicated by relaxation time *τ*; Ravindran et al. 2023; Gordon et al. 2025) showed that these mutations decreased droplet fusion dynamics (Figure 1E,F). Taken together, these findings suggest that ALS/FTD‐associated mutations accelerated the liquid‐to‐gel phase transition of p62 bodies.

### 2.2 ALS/FTD‐Linked Mutations Perturb Light‐Induced Intracellular Condensation of p62

**FIGURE 2:**
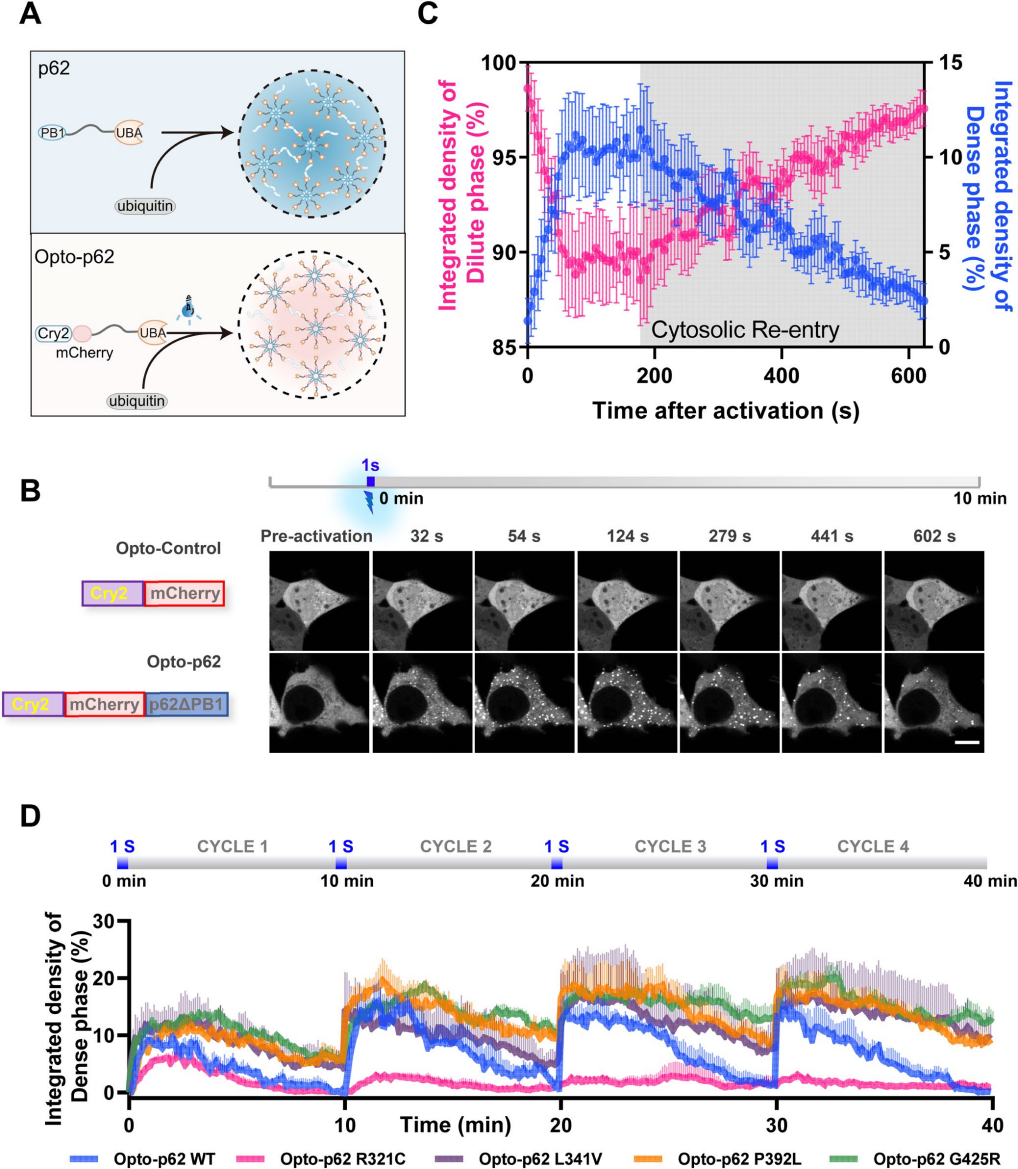
ALS/FTD‐associated p62 mutations accelerate liquid‐to‐gel phase transition of light‐induced p62 condensates. (A) Schematic representation of the p62 body formation regulated by PB1 domain or an optogenetic tool. N‐terminal PB1 domain‐driven oligomerization and the multivalent interaction between p62 and its binding partner's ubiquitin are both required for p62 condensation. (B) HEK 293 cells were transfected with Opto‐Control (Cry2 with mCherry tag) and Opto‐p62 (Cry2 with mCherry‐p62 deleted of PB1 domain) for 24 h, and time‐lapse images of condensate assembly after blue light activation and disassembly after withdrawing blue light were shown. Scale bar, 10 μm. See also Video S1. (C) The integrated fluorescence intensity of Opto‐p62 in the dense phase (condensates) and dilute phase (surrounding cytoplasm) in (B). Data are shown as mean ± SEM. (D) The integrated fluorescence intensity of Opto‐p62 (WT, R321C, L341V, P392L, or G425R) in dense phase (condensates) along with four cycles of blue light stimulation. Data are shown as mean ± SEM from three independent experiments.

According to previous studies, the PB1 domain (the N‐terminal dimerization domain) is necessary for p62 body formation (Ciuffa et al. 2015). To study the phase separation behavior of p62, we created a light‐inducible “OptoDroplet” model of p62 condensation to study “phase separation behavior,” since the previous paper showed that proteins with a self‐oligomerization domain for condensation are ideal for designing light‐inducible chimera constructs by replacing this self‐oligomerization domain with Cry2 (light‐inducible oligomerization element; Zhang et al. 2019). In detail, the PB1 domain (self‐oligomerization domain) of p62 was replaced by the blue (488 nm) light‐dependent self‐oligomerization domain (Cry2_PHR_, hereafter referred to as Cry2). We named the resulting chimeric p62 protein (Cry2‐mCherry‐p62) “Opto‐p62,” which can temporally and spatially control p62 phase separation in live cells using blue light (Figure 2A). Unlike negative Opto‐Control (Cry2‐mCherry tag alone), Opto‐p62 dramatically underwent phase separation and formed p62 body‐like droplets (p62 condensates) after short‐term blue light stimulation, and p62 bodies could spontaneously disassemble after blue light withdrawal (Figure 2B,C, Video S1). These results indicate that Opto‐p62 is an effective and highly controllable tool for studying p62 phase separation.

As presented above, the liquid‐like feature of the p62 bodies is impaired by ALS/FTD‐related mutations, and we next explored the effect of ALS/FTD‐related p62 mutations on phase separation using Opto‐p62. To this end, we fused each p62 mutation protein (deletion of PB1 domain) to Cry2, and then intermittently photo‐activated Opto‐p62 using blue light to imitate chronic and intermittent assembly and disassembly of p62 condensates in live cells (Figure 2D, Figure S2A, Video S2). The results showed that the R321C mutation inhibited the formation of p62 condensates relative to WT and other mutations, indicating the R321C mutant displays a lower phase separation tendency. Moreover, we found that other p62 mutations weakened the ability of p62 condensates to disassemble during four stimulation cycles, indicating these light‐induced condensates containing p62 mutants display gel‐like behaviors, similar to “aging‐related” pathological protein (p62, TDP‐43, and FUS) aggregates in ALS/FTD (Figure 2D, Figure S2A, Video S2; Ling et al. 2013; Patel et al. 2015).

### 2.3 Optogenetic Control of p62‐Ub Condensates in Cells

**FIGURE 3:**
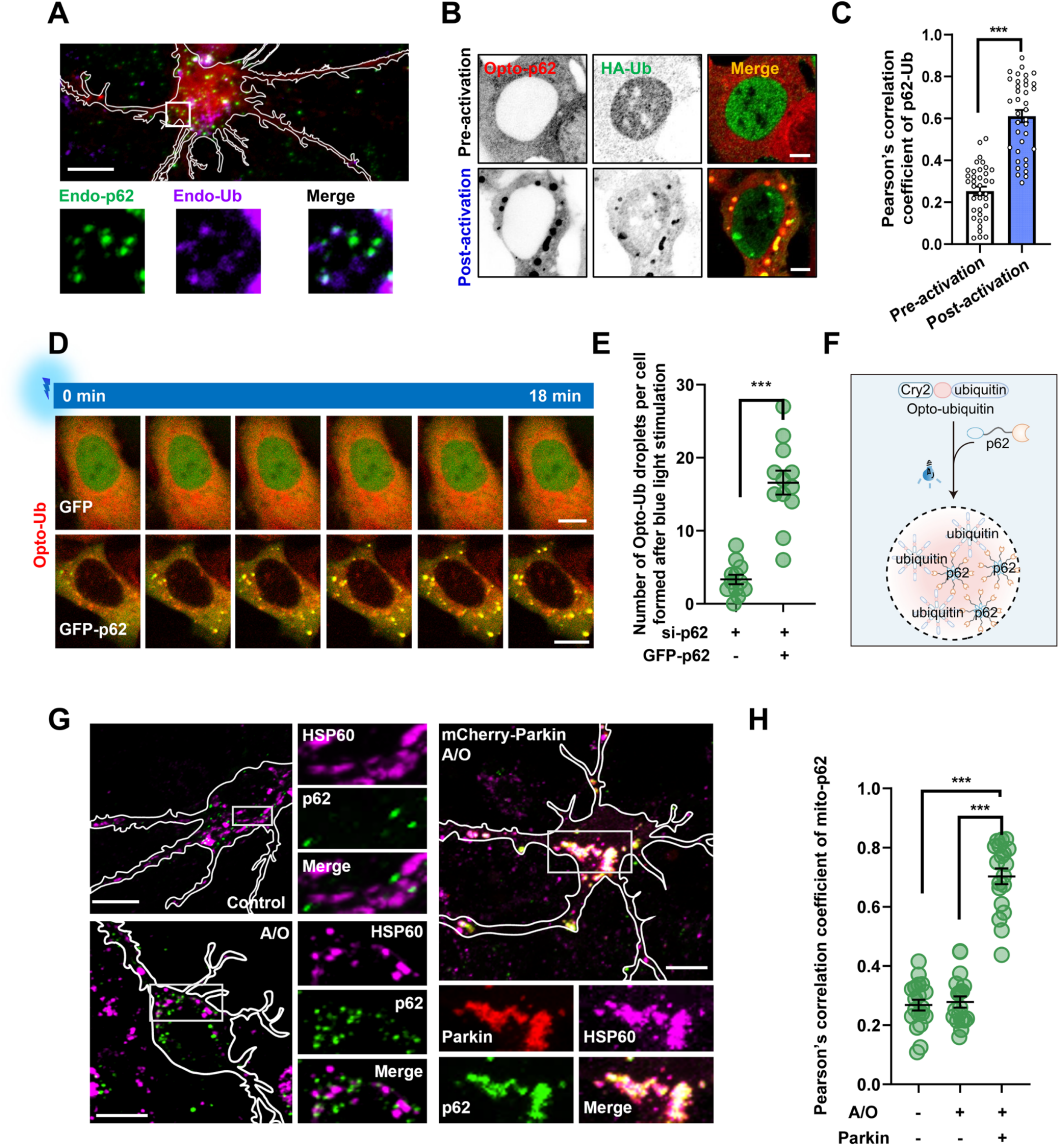
Optogenetic control of p62‐Ub condensates in cells. (A) Primary cultured mouse cortical neurons were subjected to immunofluorescent assay of endogenous (Endo) p62 and Ub using antibodies. Scale bar, 10 μm. (B) HEK 293 cells were transfected with Opto‐p62 and HA‐Ub for 24 h, and then were treated with or without blue light. The cells were fixed and stained with HA antibody using 488 nm fluorescent secondary antibody. Scale bar, 5 μm. (C) Pearson's correlation coefficient for (B). Data are shown as mean ± SEM from three independent experiments. ***p < 0.001, *t*‐test. (D) HEK 293 cells were transfected with human p62‐targeting siRNA for 48 h, and then were re‐transfected with Opto‐Ub (Cry2 with mCherry‐Ub) with GFP‐vector or GFP‐p62 for 24 h. The cells were stimulated by continuous blue light. Scale bar, 10 μm. (E) Quantification of condensate number after blue light stimulation in (D). Data are shown as mean ± SEM from three biological replicates. ***p < 0.001, *t*‐test. (F) Schematic representation of Opto‐Ub and p62 co‐condensate formation. (G) Primary cultured mouse cortical neurons were infected with or without mCherry‐Parkin lentivirus for 48 h, and then were treated with or without 5 μg/mL A/O for 4 h. The neurons were subjected to immunofluorescent assay using anti‐p62 and anti‐HSP60 antibodies. Scale bar, 10 μm. (H) Pearson's correlation coefficient of endo‐p62 and endo‐HSP60 for (G). Data are shown as mean ± SEM from three independent experiments. ***p < 0.001, one‐way Anova followed by post hoc Tukey's tests.

Given the fact that the multivalent interactions between p62 and Ub drive p62 phase separation, which in turn helps to capture ubiquitinated cargos for the recognition by ATG8 family protein and subsequent autophagy (Sun et al. 2018; Zaffagnini et al. 2018; Isogai et al. 2011), and our observation that the co‐condensation of p62 and Ub in cultured neurons (Figure 3A), we wondered whether optical control of Opto‐p62 can affect condensation of ubiquitinated proteins, and vice versa. Our results showed that photo‐activation of Opto‐p62 could induce condensate formation of ubiquitinated proteins (Figure 3B,C). In addition, we created a light‐inducible model of Ub condensation by fusing mCherry‐Ub to Cry2, named Opto‐Ub (Cry2‐mCherry‐Ub), as shown in the previous paper to study the association between Ub and p62 (Hsieh and Yang 2019). We found that p62, unlike the negative control, could co‐condense with Opto‐Ub upon photo‐activation, and decrease the condensation threshold of Opto‐Ub (Figure 3D–F). Taken together, these results indicate that p62 and ubiquitinated proteins can positively cooperate with each other to form p62‐Ub condensates, which may display potential cellular functions in the regulation of autophagy.

To explore the potential contribution of p62‐Ub condensation to mitophagy, we used the combination of Antimycin A and Oligomycin A (A/O) treatments, which can abolish mitochondrial respiration to induce PINK1/Parkin‐mediated mitophagy in cultured cells (Yang et al. 2016). We found that the Parkin C431F mutation, a mutation in the RING2 domain that abolishes Parkin E3 ligase activity and Parkin‐mediated ubiquitination of mitochondria during mitophagy (Sun et al. 2022), strikingly impaired the translocation of p62 onto A/O‐treated damaged mitochondria (Figure S2B). Importantly, we also found that p62 failed to translocate onto mitochondria in cultured neurons with healthy mitochondria (Control group) or low levels of Parkin‐mediated ubiquitination (A/O group), compared with Parkin‐expressing neurons (Figure 3G,H). These data demonstrate that the remarkable translocation of p62 onto mitochondria depends on Parkin‐mediated ubiquitination, suggesting a potential role of p62‐Ub condensation in Parkin‐mediated mitochondrial quality control.

### 2.4 p62 Condensation Modulates Damaged Mitochondrial Clustering to Inhibit Their Clearance

**FIGURE 4:**
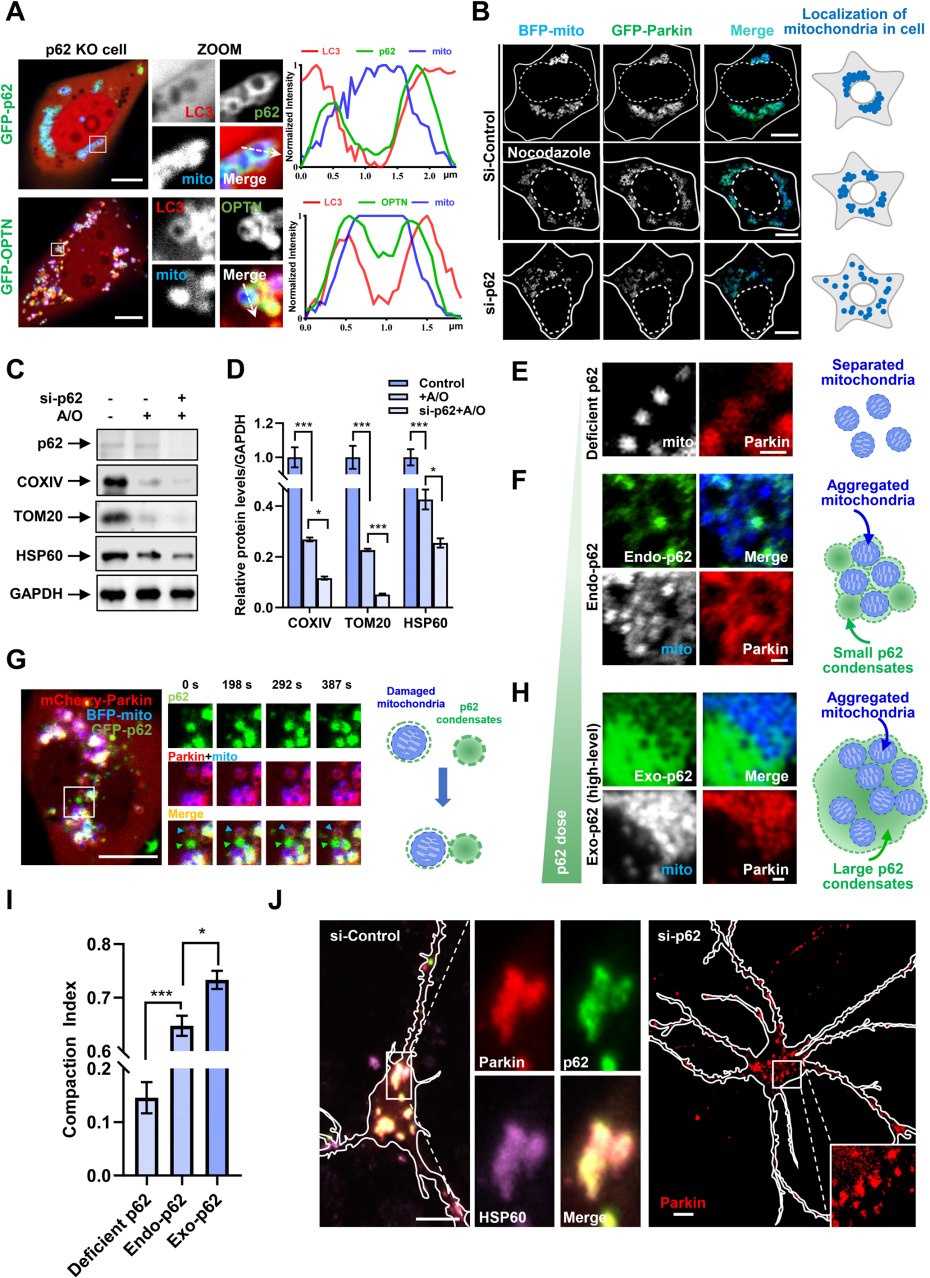
p62 condensation mediates mitochondrial clustering and inhibits mitochondrial clearance. (A) p62 knockout U2OS cells were transfected with FLAG‐Parkin, mCherry‐LC3, BFP‐mito, as well as GFP‐p62 (top) or GFP‐OPTN (bottom) for 24 h, and then were treated with 5 μg/mL A/O for 4 h. The line scan shows the fluorescence intensity of LC3, mito, and OPTN or p62. Scale bar, 10 μm. (B) HEK 293 cells were transfected with control (non‐targeting oligonucleotide) or p62 siRNA, followed by transfection with GFP‐Parkin and BFP‐mito for 24 h, and then the cells were treated with 5 μg/mL A/O for 4 h with or without the combination of nocodazole (5 μg/mL, pre‐treated for 2 h). The right is the schematic representation of mitochondrial distribution. Scale bar, 10 μm. (C) HEK 293 cells were transfected with control or p62 siRNA for 24 h, and then were re‐transfected with FLAG‐Parkin for another 24 h. After treatment with or without 1 μg/mL A/O for 16 h, the cells were collected and the cell lysates were subjected to immunoblot with indicated antibodies. Data from three independent experiments are represented. (D) Quantification of the relative protein level in (C). Data are shown as mean ± SEM from three independent experiments. *p < 0.05, ***p < 0.001, one‐way Anova followed by post hoc Tukey's tests. (E) HEK 293 cells were transfected with p62 siRNA for 48 h to generate p62‐deficient cells, followed by transfection with mCherry‐Parkin and BFP‐mito for 24 h, and then were treated with 5 μg/mL A/O for 3 h. Scale bar, 1 μm. (F) HEK 293 cells were transfected with mCherry‐Parkin and BFP‐mito for 24 h and treated with 5 μg/mL A/O for 3 h. The cells were fixed and stained with p62 antibody for detecting endogenous (Endo) p62 using 488 nm fluorescent secondary antibody. Scale bar, 1 μm. (G) Time‐lapse images of GFP‐p62, mCherry‐Parkin, and BFP‐mito in HEK 293 cells expressing p62 siRNA after treatment with 5 μg/mL A/O for 1 h. Scale bar, 10 μm. (H) HEK 293 cells were transfected with exogenous (Exo) mCherry‐p62, GFP‐Parkin, and BFP‐mito for 24 h, and then were treated with 5 μg/mL A/O for 3 h. Scale bar, 1 μm. (I) Compaction index (the formula of compaction index is in the experimental procedures) for mitochondria in Endo‐p62 (F) and Exo‐p62 (H) condensates. Data are shown as mean ± SEM. *p < 0.05, one‐way Anova followced by post hoc Tukey's tests. (J) Primary cultured mouse cortical neurons were infected with mCherry‐Parkin lentivirus and transfected with control or mouse p62‐targeting siRNA for 48 h, and then the neurons were treated with 5 μg/mL A/O for 4 h. The neurons in si‐Control group were subjected to immunofluorescence staining assay using anti‐p62 and anti‐HSP60 antibodies. Scale bar, 10 μm.

To decipher the special role of different autophagy receptors in the quality control of mitochondria, we carefully examined the contribution of p62 and OPTN to PINK1/Parkin‐mediated mitophagy in cells. Notably, we observed that p62 tended to associate with “grape‐like” mitochondrial clustering (Hsieh and Yang 2019), whereas OPTN colocalized with LC3 on dispersed mitochondria in p62 knockout cells (Figure 4A, Figure S2C), indicating that p62 depletion is associated with dispersed distribution of mitochondria in PINK1/Parkin‐mediated mitophagy. To further explore the special mechanism of mitochondrial clustering, we first tested the effect of the microtubule network (regulator of mitochondrial positioning) and p62 on mitochondrial clustering. In contrast to perinuclear “grape‐like” clusters after 4 h of A/O treatment, we found that disassembly of microtubule scattered grape‐like mitochondrial clusters to smaller mitochondrial clusters, whereas p62 depletion drove complete dispersion of mitochondria in the cytosol (Figure 4B, Figure S2D,E). These results indicate that p62 condenses damaged mitochondria to form mitochondrial clusters with small size, and the microtubule network stretches mitochondria clusters together to form integrated mitochondrial clusters (the clustering of depolarized mitochondria within a juxtanuclear region, compared with simple p62 phase separation‐mediated mitochondrial clusters). To assess the functional significance of p62 in mitophagy, we examined whether damaged mitochondria could still be degraded in p62‐depleted cells. The results showed that knockdown of p62 increased the process of mitochondrial clearance after A/O treatment (Figure 4C,D).

To further explore the relationship between membrane‐free p62 condensation and mitochondrial clustering, we evaluated the p62‐associated role with different expression levels in mitochondrial clustering during mitophagy. Surprisingly, in contrast to dispersive mitochondria in p62‐depleted cells (Figure 4E), we found that endogenous p62 condensates (puncta structures not autophagosomes) stuck to p62‐labeled mitochondria (Figure 4F), where p62 condensates acted as a “bridge” for linking individual mitochondria at the incipient stage of mitophagy in cells (Figure 4G).

Furthermore, we found high expression level of p62 could form a giant condensate which contained multiple mitochondria inside the body during mitophagy (Figure 4H), and quantification data suggest that the clustering of mitochondria (as indicated by compaction index [CI]; Narendra et al. 2010) was enhanced by the expression level of p62 (Figure 4I). In addition, we showed that p62 depletion impaired the formation of perinuclear “grape‐like” clusters in mouse primary neuronal culture (Figure 4J, Figure S2D) and human neuronal SH‐SY5Y cells (Figure S3A). Our data indicate that p62 condensation is both necessary and sufficient for mitochondrial clustering during mitophagy.

### 2.5 p62 Phase Separation Is the Driving Force of Mitochondrial Clustering During Mitophagy

**FIGURE 5:**
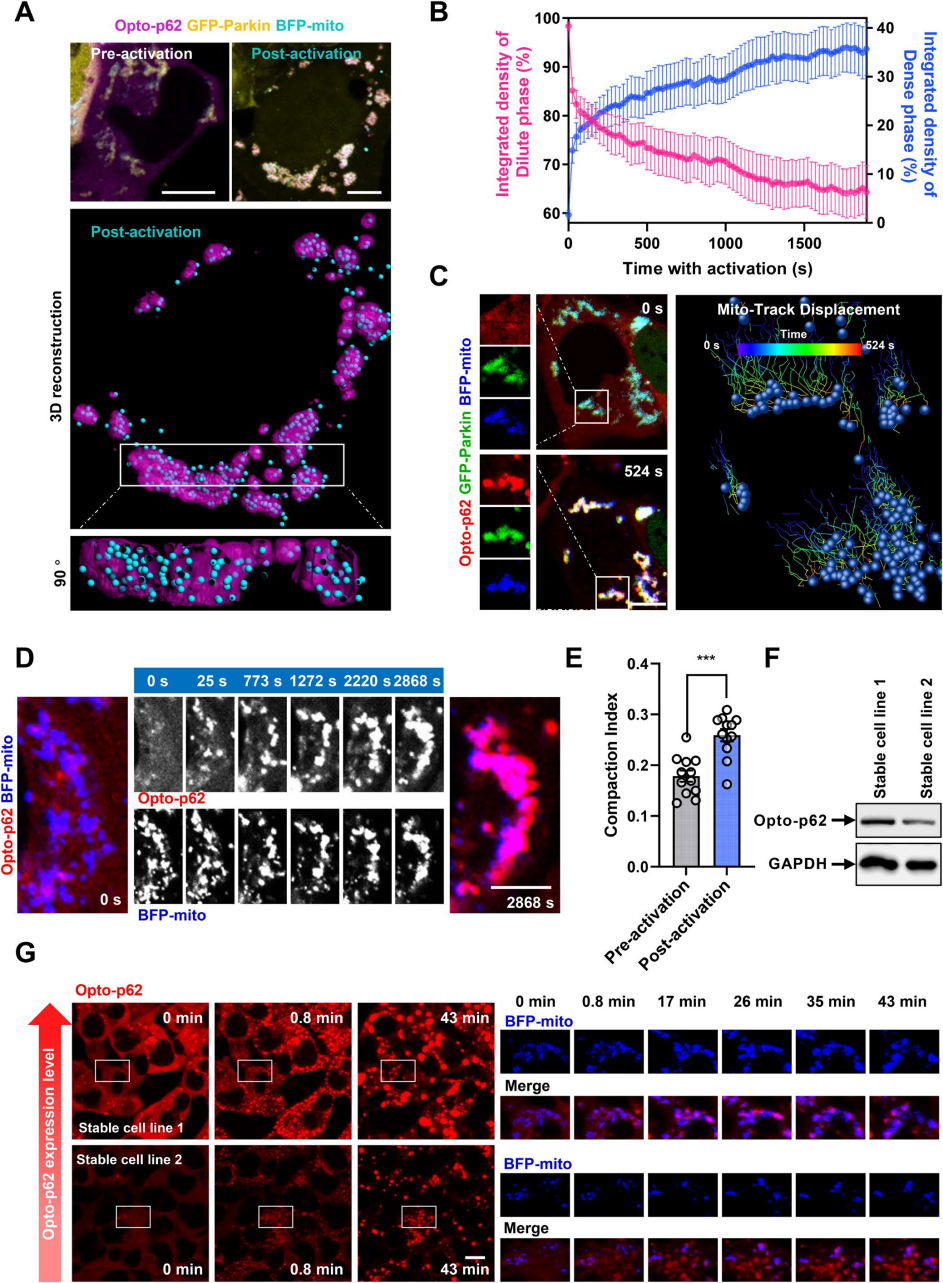
p62 phase separation directly promotes mitochondrial clustering. (A) p62‐depleted HEK 293 cells were transfected with Opto‐p62, BFP‐mito and GFP‐Parkin for 24 h, and then the cells were treated with 5 μg/mL A/O for 2 h. Upper panels: Representative fluorescence images; lower panels: 3D reconstruction of p62 condensates and mitochondria for post‐activated cell image. (B) The integrated fluorescence intensity of Opto‐p62 in the dense phase (condensates) and dilute phase (surrounding cytoplasm) in (A). Data are shown as mean ± SEM. (C) Similar experiments as in (A). Right panels: Corresponding mitochondrion displacement (dot, mitochondria at 524 s; line, mitochondrial motion trail) during the 524 s. Scale bar, 10 μm. (D) p62‐depleted HEK 293 cells were transfected with Opto‐p62, BFP‐mito, and HA‐Parkin for 24 h. After 1.5 h A/O treatment, the cells were stimulated by blue light. Scale bar, 5 μm. (E) Compaction index for mitochondria in (D) pre‐ (0 s) and post‐ (2868 s) blue light stimulation. Data are shown as mean ± SEM. ***p < 0.001, *t*‐test. (F) The cell lysates of SH‐SY5Y cells stalely expressing Opto‐p62, were subjected to immunoblot with indicated antibodies. (G) SH‐SY5Y cells, which were stalely expressing Opto‐p62, were transfected with human p62‐targeting siRNA, and then were re‐transfected with BFP‐mito and GFP‐Parkin for 24 h. After 1.5 h A/O treatment, the cells were stimulated by blue light. Scale bar, 10 μm.

To confirm the direct role of p62 phase separation on mitochondrial clustering, we analyzed the relationship between light‐induced p62 phase separation and mitochondrial distribution in p62‐depleted cells expressing Opto‐p62 (Figure 5A), which could dramatically phase separate into dense phase on damaged mitochondria from dilute phase (cytosol) upon stimulation (Figure 5B). As shown in 3D reconstruction, upon blue light‐induced phase separation, condensed p62 strongly colocalized with mitochondria and promoted mitochondrial clustering (Figure 5A). Furthermore, the mitochondrial track displacement program indicates that separated mitochondria tend to be compacted together into smaller regions along with activating blue light (Figure 5C). To explore the dynamic process of p62 condensation‐mediated mitochondrial clustering, we used time series modeling of live cell imaging and found that blue light‐induced p62 condensates dramatically condensed onto mitochondria and promoted mitochondrial clustering (Figure 5D). Quantification data further suggest that the CI of mitochondria (Narendra et al. 2010) was enhanced by light‐induced p62 condensation (Figure 5E). Furthermore, we established various SH‐SY5Y cell lines stably expressing Opto‐p62, and found a positive correlation between Opto‐p62 expression level and induction of Opto‐p62 condensates (Figure 5F,G). Importantly, we found that light‐induced p62 condensation significantly led to the clustering of mitochondria, even at relatively low Opto‐p62 expression level (Figure 5G). Along with the evidence that p62 condensates entrap mitochondria together in a Parkin‐mediated ubiquitination‐dependent manner (Figures 3G,H and 4F,G), these data suggest that p62‐Ub multivalent interaction‐mediated condensation drives clustering of ubiquitinated mitochondria during PINK1/Parkin‐mediated mitophagy. Taken together, our data demonstrate that unlike OPTN, which mediates autophagosome recruitment onto damaged mitochondria during mitophagy, p62 plays a different role in mitochondrial quality control by triggering damaged mitochondrial clustering through phase separation.

### 2.6 ALS/FTD‐Related p62 Mutations Inhibit Mitochondrial Clustering, Resulting in Excessive Clearance of Mitochondria

**FIGURE 6:**
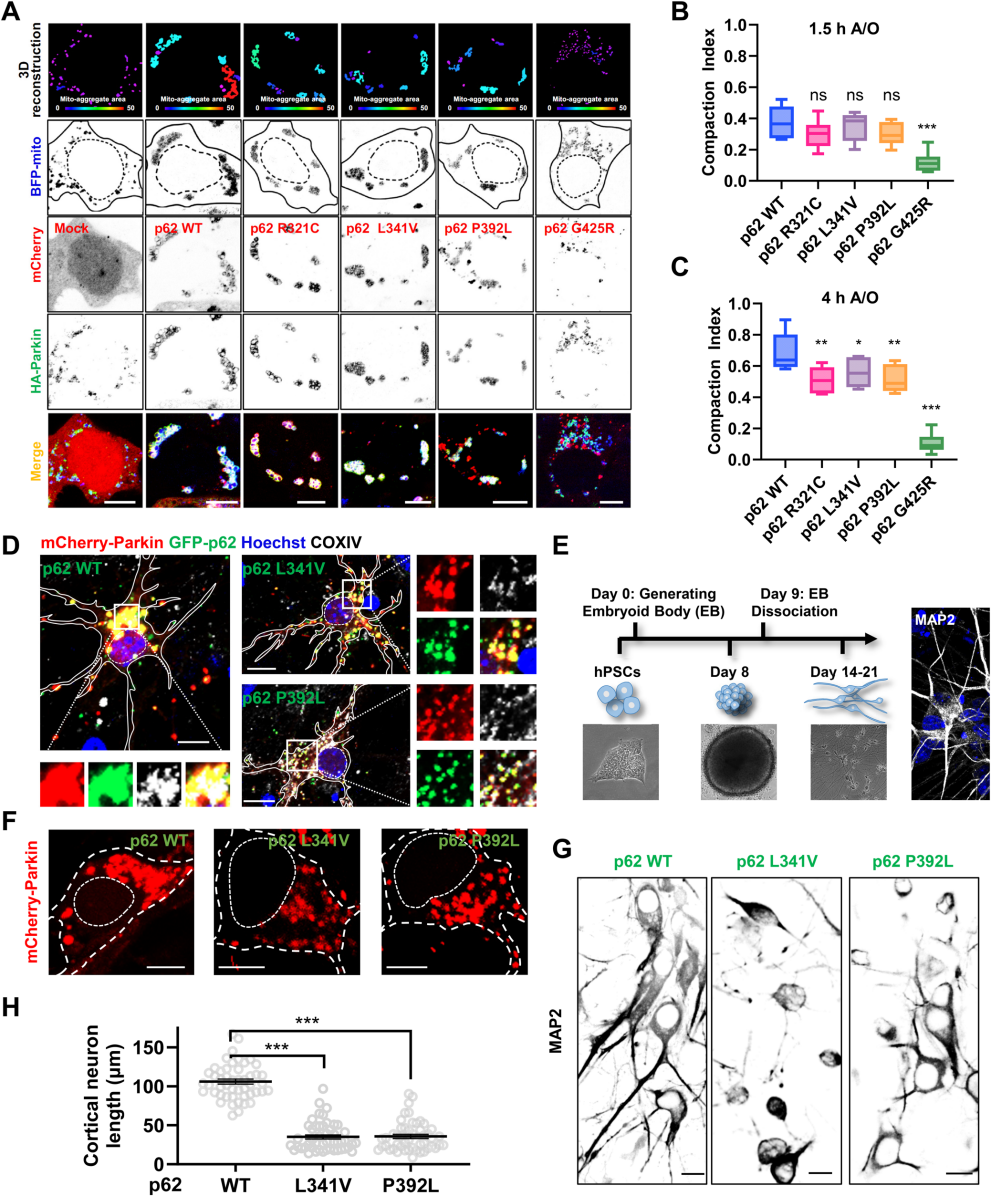
ALS/FTD‐associated mutations perturb p62‐mediated mitochondrial quality control during mitophagy. (A) p62‐depleted HEK 293 cells were transfected with mCherry‐Vector or mCherry‐p62 (WT, R321C, L341V, P392L, or G425R), HA‐Parkin and BFP‐mito for 24 h, and then were treated with 5 μg/mL A/O for 4 h. Scale bar, 10 μm. Upper panels: 3D reconstruction of mitochondrial cluster volumes. Warmer colors in the color bar indicate increasing mitochondrial clustering. (B, C) Boxplot (min–max) of compaction index for mitochondria after 1.5 h (B) or 4 h (C) A/O treatment. Data from three independent experiments are represented. *p < 0.05, **p < 0.01, ***p < 0.001; ns, not significant, one‐way Anova followed by post hoc Tukey's tests. (D) Primary cultured mouse cortical neurons were transfected with p62 siRNA, and then were infected with mCherry‐Parkin and GFP‐p62 (WT, L341V, or P392L) lentivirus for 48 h. The neurons were treated with 5 μg/mL Antimycin A and Oligomycin A (A/O) for 6 h, and then were subjected to an immunofluorescence assay using anti‐COXIV antibody and Hoechst. Scale bar, 10 μm. (E) The schematic diagram of the human iPSC differentiation procedure. Right: iNeurons were fixed and stained using anti‐MAP2 antibody and Hoechst. (F) The iNeurons were transfected with p62 siRNA, and then were infected with mCherry‐Parkin and GFP‐p62 (WT, L341V, or P392L) lentivirus for 48 h. The iNeurons were treated with 5 μg/mL A/O for 6 h, and then were subjected to imaging. Scale bar, 5 μm. (G) Primary cultured mouse cortical neurons were transfected with siRNA targeting p62, and then were infected with mCherry‐Parkin and GFP‐p62 (WT, L341V, or P392L) lentivirus for 48 h. The neurons were treated with 1 μg/mL Antimycin A for 12 h, and then were subjected to immunofluorescence assay using anti‐MAP2 antibody. Scale bar, 10 μm. (H) Quantification of the neurite length from 40 neuron cells in (G). Quantification data are shown as mean ± SEM from three independent experiments. ***p < 0.001, one‐way Anova followed by post hoc Tukey's tests.

Given the fact that ALS/FTD‐related p62 mutations disrupt p62 phase separation which regulates mitochondrial clustering and clearance in PINK1/Parkin‐mediated mitophagy as mentioned above, we wondered whether ALS/FTD‐related mutations disturb mitochondrial clustering and clearance. Importantly, G425R mutation delayed mitochondrial clustering relative to WT and other mutations after 1.5 h A/O treatment (Figure S3B). Other mutations, but not WT, also perturbed mitochondrial clustering after A/O treatment in HEK 293 cells (Figure 6A), SH‐SY5Y cells (Figure S3C), mouse primary neuron (Figure 6D) and human induced pluripotent stem cell (iPSC)‐derived neurons (iNeurons; Figure 6E,F). Moreover, the CI analysis (Narendra et al. 2010) showed the same results (Figure 6B,C), indicating these p62 mutations, especially p62 G425R mutant, inhibit mitochondrial clustering. To further validate our results using mouse models, we tried to generate AAV‐based p62 (WT and ALS/FTD mutant) mouse model (Figure S3D), and then purified p62 bodies from mouse brain tissue and combined them with purified mitochondria from p62 knockout U2OS cells (Figure S3E), an assay of which is used to assess the ability of central nervous system‐derived p62 condensates on mitochondrial clustering. The results showed that p62 WT, but not P392L mutant condensates, promoted mitochondrial clustering (Figure S3F–H). To further study the effect of p62 condensation on mitochondrial clearance, we performed a long‐term A/O treatment assay. The results showed that, compared with WT p62, p62 mutations speeded up the clearance of damaged mitochondria (Figure S4A–C). To explore the contributions of p62 pathogenic mutations to neurotoxicity upon mitochondrial damage, we used chronic and mild Antimycin A treatment to imitate chronic mitophagy and disease environment and used MAP2 (a neuron‐specific marker) to examine neurite outgrowth. The results showed that ALS/FTD‐associated p62 pathogenic mutations, which impair mitochondrial clustering, reduce neurite outgrowth in primary cultured neurons upon mitochondrial damage (Figure 6G,H), indicating p62‐mediated mitochondrial clustering display protective role upon mitochondrial damage‐triggered mitophagy. Together, these results suggest that ALS/FTD‐related p62 mutations inhibit mitochondrial clustering and then speed up mitochondrial clearance, resulting in dysfunctional mitochondrial quality control.

## 3 Discussion

In mitophagy, damaged mitochondria are first ubiquitinated by PINK1/Parkin and gather together around the nucleus to form “grape‐like” mitochondrial clusters, which are then remodeled to release free‐floating mitochondria in an F‐actin‐dependent manner (Hsieh and Yang 2019). However, the specific mechanism and pathophysiological significance of the assembly of “grape‐like” mitochondrial clusters during mitophagy remain elusive. Interestingly, unlike other essential autophagy receptors (OPTN and NDP52) in mitophagy, p62 depletion cannot delay mitophagy (Wong and Holzbaur 2014), but impedes perinuclear mitochondrial clustering (Okatsu et al. 2010). Along with our findings on p62 phase separation, it is likely that the process of p62‐mediated mitochondrial clustering avoids over‐degradation of mitochondria. Therefore, p62 is unnecessary for presenting these ubiquitinated mitochondria to autophagosomes (Lazarou et al. 2015), but its phase separation plays an important role in mitochondrial quality control during PINK1/Parkin‐mediated mitophagy.

Notably, with the help of a spatiotemporally optogenetic tool, we demonstrate that biomolecular condensation of p62 directly drives mitochondrial clustering to form grape‐like clusters (Figure 5). We also demonstrate that p62 condensation‐mediated mitochondrial clustering can be driven by the interaction between p62 and poly‐Ub at physiological level in cells and in neurons (Figures 3G and 4). Relevant to this, a recent study also showed that light‐induced p62 condensation drives the clustering of small polyQ aggregates together (Choi et al. 2024), further suggesting that p62 may regulate clustering of various cellular compartments (including proteins and organelles) through phase separation. Interestingly, other autophagy receptors, OPTN, NDP52, and TAX1BP1, also contain oligomerization domain (Li et al. 2016; Richter et al. 2016; White et al. 2023), but these oligomerization domains are associated with coiled‐coil (CC) region, therefore they may vary from the PB1 domain in p62, leading to different condensation potential of these autophagy receptors. It is possible that these autophagy receptors also mediate substrate‐associated condensation to regulate other forms of selective autophagy. In the future, it will be worth studying the roles of condensation of these autophagy receptors in organelle quality control.

Interestingly, we also find the large perinuclear mitochondrial clusters disperse to several approximately round and smaller mitochondrial clusters after microtubule‐depolymerizing drug treatment, suggesting that microtubule may slightly medicate retrograde transport of mitochondrial clusters, whereas p62 plays an essential role in mitochondrial clustering (Figure 4B). Note that the effect of high‐level expressed p62 may vary from endogenous (low‐level) p62 on condensation and mitochondrial clustering, but towards the same tendency and nature. Relevant to this, we used stable cell lines expressing low‐level Opto‐p62, and found that after blue light stimulation, p62 condensates partially wetting on mitochondria and induces mitochondrial clustering (Figure 5G), similar to endogenous p62‐mediated partial wetting and clustering (Figure 4F,G). Taken together, our data suggest that the p62 protein level affects the degree of wetting effect on mitochondria, therefore influencing mitochondrial clustering—the higher protein level results in a stronger effect.

Another notable finding of the present study is that we showed this mitochondrial clustering process is a balancing mechanism driven by p62 condensation to avoid fast turnover of mitochondria in cells (Figure 4). There are two possible scenarios for what would happen after p62‐mediated mitochondrial clustering: (1) The mitochondrial cluster can be degraded by autophagy—but at a relatively slower rate, like the autophagy degradation of aggresome formed by misfolded proteins (Johnston et al. 1998; Kopito 2000; Chin et al. 2010; Wang et al. 2012); (2) the cells utilize p62 to deposit a large number of damaged mitochondria together, and then use a “secretion” way to release single/free mitochondria for OPTN‐mediated degradation. In this scenario, phase separation of p62 can drive the “condensation” of damaged mitochondria to form an “aggresome”‐like cluster of mitochondria during mitophagy—it is possible that the surface of mitochondria is enriched with condensed p62 which limits the “space” for OPTN to occupy at this stage. Therefore, this mitochondrial clustering process can be considered as an arrest mechanism driven by p62 (which is far beyond the function of other autophagy receptors in mitophagy such as OPTN) to avoid fast turnover of mitochondria mediated by OPTN in cells. On the other hand, free mitochondria detached from “p62‐enriched mitochondrial aggresome” can be occupied by OPTN and further processed for autophagic degradation, and this process can be considered as an ER‐Golgi secretion‐like sorting pathway to govern mitochondrial quality control.

Importantly, the multivalent interactions between ALS proteins (such as TDP‐43, FUS, and hnRNPA1) and RNA will lead to liquid‐to‐liquid phase separation. Under disease or stress conditions, these proteins will undergo pathological liquid‐to‐solid phase transition, which can be considered as a toxic gain of function mechanism (Patel et al. 2015; Molliex et al. 2015; Mann et al. 2019; Alberti and Hyman 2021). Although multivalent interactions between p62 and poly‐Ub similarly drive phase separation and the condensation‐mediated mitochondria clustering process, ALS/FTD‐associated p62 mutations impair this process, indicating that loss of function mechanism may at least partially contribute to p62‐mediated ALS/FTD disease pathogenesis. Related to a recent report that investigated the fluidity of condensates containing several p62 pathogenic mutants (Faruk et al. 2021), we showed these ALS/FTD‐linked p62 mutations (Fecto et al. 2011; Chen et al. 2014) reduce the liquidity of p62 bodies (condensates; Figure 1) and phase separation “tendency” (the ability to form condensates; Figure 2), therefore producing unhealthy pathological condensates. In these mutations, P392L and G425R mutations all exist in the Ub‐binding UBA domain, which may reduce the binding affinity of poly‐Ub to p62; therefore, we speculate that these p62 mutations may not affect phase separation of p62 per se, but can impair phase separation by affecting the p62‐Ub multivalent interaction (Sun et al. 2018). It is interesting that R321C and L341V mutations exist in an intrinsically disordered region (IDR)‐like non‐folding region (Komatsu 2022). Although previous studies showed that mutations in IDR (a non‐folded region that typically displays phase separation potential) can lead to the alteration of protein phase separation, it is unlikely that R321C and L341V mutations directly affect p62 condensation in cells since the multivalent interaction between p62 and poly‐Ub is the major driving force of p62 condensation. Therefore, R321 and L341 mutations may indirectly influence p62 condensation by affecting p62‐Ub interaction.

In addition to the contribution of p62‐mediated mitochondrial clustering to ALS/FTD pathogenesis, it is possible that p62‐mediated condensation also plays important roles in other neurodegenerative diseases such as PD and AD. In detail, PD‐linked PINK1/Parkin, which promotes mitochondrial ubiquitination, acts as an upstream regulator in mitophagy; whereas ALS/FTD‐linked p62 and OPTN act downstream of PINK1/Parkin‐mediated ubiquitination—it is very important that the downstream p62‐mediated condensation may affect the entire mitochondrial quality control machinery and contribute to the pathogenesis of both PD and ALS, and possibly other aging‐related neurodegenerative diseases such as AD. Moreover, OPTN loss of function pathogenic mutation leads to defects in mitophagy, which may contribute to disease pathogenesis, whereas p62 ALS/FTD mutations trigger impairment of mitochondrial clustering during mitophagy, which can be considered as loss of function—it may combine with the toxic gain of function effects triggered by abnormal pathological phase transition of p62 mutants, collectively contributing to the disease pathogenesis.

Taken together, our findings reveal that phase‐separated p62 supplies a mitochondrial “storage pool” by clustering damaged mitochondria to avoid the over‐degradation of mitochondria as a self‐balanced mitochondrial quality control machinery. The present study provides novel insight into the mechanism by which membrane‐less condensate controls membrane‐bound organelles to maintain cellular homeostasis. Importantly, ALS/FTD‐linked mutations perturb p62 condensation and mitochondrial clustering, thereby impairing the balance of mitochondrial quality control during mitophagy.

## 4 Methods

### 4.1 Plasmid Constructs and siRNAs

EGFP‐Vector, EGFP‐p62, mCherry‐LC3, BFP‐mito, EGFP‐OPTN, HA‐Parkin, FLAG‐Parkin, mCherry‐Parkin, GFP‐Parkin, HA‐Ub, pET‐15b, and mt‐Keima plasmids were described previously (Sun et al. 2022; Zhou et al. 2013). Lentiviral packaging plasmids were kindly provided by Qian Li (Shanghai Jiao Tong University). mCherry‐C1‐p62 was created by subcloning PCR product with BglII/EcoRI sites from EGFP‐N3‐p62 into mCherry‐C1 vector. Cry2‐mCherry vector was created by subcloning PCR from the original plasmid containing Cry2‐mCherry. Cry2‐mCherry‐Ub was created by subcloning PCR product with XhoI/BamHI sites from HA‐Ub into the Cry2‐mCherry vector. His‐mCherry vector was created by subcloning PCR product from the mCherry vector to pET‐15b. His‐mCherry‐p62 was created by subcloning PCR product with NotI/XhoI sites from EGFP‐p62 to His‐mCherry vector. GST‐GFP‐4xUb was produced by Genscript, and GST‐GFP‐4xUb‐His was generated by homologous recombination using ClonExpress II One Step Cloning Kit (Vazyme) with the following primers: AGCGGCCATCATCATCATCATCACTGAGCGGCCGCATCGTGA and TGATGATGATGGCCGCTGCTGCTGCCGCGCGGCACCAGTCCTCCTCGTAGTCGCAGGA. Lysine mutants (R321C, L341V, P392L, G425R) of Cry2‐mCherry‐tagged, mCherry‐C1‐tagged, and EGFP‐C1‐tagged p62 were generated by homologous recombination using ClonExpress II One Step Cloning Kit (Vazyme) with the following primers: 5′‐TGCCCTGAGGAACAGATGGAGTCGGATAACTG‐3′ and 5′‐ATCTGTTCCTCAGGGCACCCCTCGGACTCCAAGGC‐3′ for R321C mutant; 5′‐GACCCATGTGTCTTCAAAAGAAGTGGACCCGT‐3′ and 5′‐TTGAAGACACATGGGTCCAGTCATCATCTCCTC‐3′ for L341V mutant; 5′‐CTGACCTGCGGCTGATTGAGTCCCTCTCCCAG‐3′ and 5′‐AATCAGCCGCAGGTCAGCCTCTGGCGGGAGAT‐3′ for P392L; 5′‐ATGACATCAGAGCGGCTCTGGACACCATCCAG‐3′ and 5′‐AGCCGCTCTGATGTCATAGTTCTTGGTCTGCAGG‐3′ for G425R mutant. Cry2‐mCherry‐p62 (Δ1–122) was created by homologous recombination from EGFP‐N3‐p62 to Cry2‐mCherry vector with the following primers: 5′‐CTGTACAAGCACCCCAATGTGATCTGCGA‐3′; 5′‐ATTGGGGTGCTTGTACAGCTCGTCCATGCC‐3′; 5′‐TTGTGATCCGGACTCAGATCTCGAGAAG‐3′; 5′‐TCTGAGTCCGGATCACAACGGCGGGGGATG‐3′. Lentivirus‐Cry2‐mCherry‐p62 (Δ1–122) was created by homologous recombination from Cry2‐mCherry‐p62 (Δ1–122) to pHAGE‐MCS‐3 × FLAG vector with BamHI/XhoI sites with the following primers: 5′‐TCGGGTTTAAACGGATCCATGAAGATGGACAAAAAGACC‐3′; 5′‐GGGCCCTCTAGACTCGAGTCACAACGGCGGGGGATG‐3′. Lentivirus‐GFP‐p62‐WT, Lentivirus‐GFP‐p62‐L341V and Lentivirus‐GFP‐p62‐P392L were created by homologous recombination from GFP‐p62‐WT, GFP‐p62‐L341V, and GFP‐p62‐P392L to pHAGE‐MCS‐3 × FLAG vector with BamHI/XhoI sites with the following primers: 5′‐TCGGGTTTAAACGGATCCATGGTGAGCAAG‐3′; 5′‐GGGCCCTCTAGACTCGAGTCACAACGGCGG‐3′.

The following siRNAs (GenePharma) were used: Negative control: A non‐targeting oligonucleotide; Human p62: 5′‐CAUGUCCUACGUGAAGGAUGATT‐3′; Mouse p62: 5′‐GGAACUCGCUAUAAGUGCATT‐3′.

### 4.2 Protein Purification In Vitro

His‐mCherry‐p62 and GST‐GFP‐4xUb‐His were expressed and purified from BL21 *Escherichia coli* cells, respectively. After being induced by IPTG at 16°C overnight, cells were then centrifuged and resuspended in lysis buffer (20 mM Tris–HCl, 500 mM NaCl, 20 mM imidazole, 5% Glycerol, 0.5 mM PMSF, 2 mM βME) and then the resuspension solution was sonicated and pelleted at 10,000 *g* at 4°C for 40 min. The supernatant was added onto Ni Sepharose FF (Vazyme), and then washed column with lysis buffer. After eluted with imidazole (250 mM), the proteins were cleaved by thrombin (Solarbio) overnight at 4°C to cleave His tag and further purified and filtered by Amicon Ultra‐0.5 (Merck). Finally, the proteins were stored in the stock buffer (500 mM NaCl, 50 mM Tris–HCl, 2 mM DTT) at −80°C and analyzed by SDS‐PAGE.

### 4.3 Cell Culture, Transfection, Lentivirus Harvest and Concentration, and Drug Treatment

Human embryonic kidney 293 (HEK 293) cells, HEK 293T, SH‐SY5Y, Neuro‐2a, and U2OS cells were cultured in 90% Dulbecco modified essential medium (DMEM; Gibco, 11995500), 10% fetal bovine serum (FBS; Gibco, 10099141C), 100 μg/mL streptomycin, and 100 U/mL penicillin (Gibco, 15140122). The p62 knockout U2OS cell lines mediated by CRISPR‐Cas9 were kindly provided by Yanfen Liu (ShanghaiTech University).

The plasmids were wrapped by Lipofectamine 2000 reagent (Invitrogen, 11668019) in Opti‐MEM (Gibco, 31985070) without serum medium for 15 min, and then transfected into the HEK 293 cells according to the manufacturer's instructions. The Opti‐MEM containing plasmids was added to cultured cells after discarding the culture medium, and the culture medium was added to these cells after 4 h. After transfection for 24 h, the cells were treated with drug or observed. The siRNA was wrapped by RNAiMAX transfection reagent (Invitrogen) in Opti‐MEM (Gibco, 31985070) for 15 min, and then transfected into the cells upon splitting according to the manufacturer's instructions.

The preparation of primary mouse cortical neurons was previously described (Xia et al. 2016). Briefly, cortical neurons were obtained from the cortex of C57BL/6 mouse embryos at embryonic Day 17. The dissociated cortical neurons were cultured in a neurobasal medium (Gibco, 21103049) with 10% FBS for 8 h, and then the medium was replaced with a supplemented neurobasal medium without serum. After being cultured for 7–9 days, the neurons were used in experiment.

The human iPSCs (National Collection of Authenticated Cell Cultures) were cultured in mTeSR1 medium (StemCell Technologies, 85850) supplemented with 10 μM ROCK inhibitor Y‐27632 (MedChemExpress) on Matrigel‐coated dishes. For neuronal differentiation modified by STEMdiff Differentiation Kit, iPSCs were dissociated with Accutase (Corning) and centrifuged at 300 *g* for 5 min. The iPSCs were seeded into 96‐well ultra‐low attachment plates (1 × 10^5^ cells/well) and cultured in Differentiation Medium to form embryoid bodies. After 9 days, the embryoid bodies were dissociated and cultured in Differentiation Medium to form iNeurons. After being cultured for 14–21 days, the iNeurons were used in experiment. The establishment of Opto‐p62‐expressing monoclonal cell lines was described previously (Luo et al. 2023). Briefly, lentivirus was produced with HEK 293T cells transfected with psPAX2 (packaging plasmids), pMD2.G (envelope plasmid), and Virus‐Cry2‐mCherry‐p62 (Δ1–122; transfer plasmid) by using Lipofectamine 2000. After 24 and 48 h, the viral supernatant in HEK 293T cells was respectively collected and filtered by a 0.45‐μm filter unit (Millipore), and then the viral supernatant along with 8 μg/mL polybrene (HanBio Biotechnology) was added to cultured SH‐SY5Y cells for 24 h. These cells were diluted into 96‐well plates (one‐cell‐per‐well), observed under a microscope after 7 days, and selected and cultured to establish monoclonal cell lines.

The procedure of Lentivirus‐GFP‐p62‐WT, Lentivirus‐GFP‐p62‐L341V, or Lentivirus‐GFP‐p62‐P392L was similar to that mentioned for the production of Lentivirus‐Opto‐p62. Then the harvested lentiviruses were concentrated by ultracentrifugation (25,000 rpm for 2 h at 4°C) and resuspended using 1xPBS. Finally, the viruses were stored at −80°C. Lentivirus‐mCherry‐Parkin was from HanBio Biotechnology.

The following chemicals were used for treatment: nocodazole (5 μg/mL; MedChemExpress), Antimycin A (1 or 5 μg/mL; Sigma, A8674), and Oligomycin A (1 or 5 μg/mL; Selleck Chemicals, S1478).

### 4.4 Mitochondrial Isolation

Mitochondrial isolation was previously described (Sun et al. 2022). Briefly, the cells were harvested in mitochondrial separation reagent A with PMSF (Beyotime Biotechnology) and ground using a Dounce tissue grinder (KIMBLE). The homogenates were centrifuged at 600 *g* for 10 min at 4°C to get supernatant total proteins, and then the supernatant was centrifuged at 11,000 *g* for 10 min at 4°C to get supernatant cytosolic proteins. The isolated mitochondria in sediment were resuspended using stock buffer (500 mM NaCl, 50 mM Tris–HCl, 2 mM DTT). Finally, the samples were analyzed by SDS‐PAGE.

### 4.5 Stereotaxic Injection and Animal Sample Preparation

The neonatal pups (C57BL/6 mice, P0) were anesthetized under ice for 5 min and then were bilaterally injected with 2 μL of AAV particles using intra‐cerebroventricular injection (ICV) at the speed of 0.2 μL/min. After the injection, the needle remained in place for 1 min, and the mice were placed in an incubator and then returned to their home cage. The AAV‐PHP.eB‐CAG > Kozak‐p62 WT‐3 × Flag‐P2A‐EGFP‐Poly(A) (titer: 2.5 × 10^13^ GC/mL) and AAV‐PHP.eB‐CAG > Kozak‐p62 P392L‐3 × Flag‐P2A‐EGFP‐Poly(A) (titer: 3 × 10^13^ GC/mL) were purchased from Cyagen Biosciences (Guangzhou, China). The experimental protocols were evaluated and approved by the Ethics and Animal Care and Use Committee of Soochow University.

The biological sample collection of brain tissues was previously described (Sun et al. 2025). Briefly, the collected mouse brains were first fixed using 4% paraformaldehyde overnight and dehydrated using 30% sucrose, and then encapsulated in OTC at −80°C and used by frozen sectioning at −20°C. In the purification experiment of p62 body in brain tissues, the collected brain samples were first homogenized in cell lysis buffer (1% NP‐40, 25 mM Tris–HCl [pH 7.6], 150 mM NaCl, and 1% sodium deoxycholate with protease inhibitor cocktail [Roche, 4693132001]). The homogenates were centrifuged at 13,000 *g* for 20 min at 4°C, and the supernatant containing p62 bodies was used to perform mitochondrial clustering experiment.

### 4.6 Immunoblot Experiment

The immunoblot experiment was previously described (Zhou et al. 2013; Lv et al. 2017; Liu et al. 2017; Yang et al. 2018; Wang et al. 2018). In detail, the processed cells were lysed to obtain proteins using cell lysis buffer (1% NP‐40, 25 mM Tris–HCl [pH 7.6], 150 mM NaCl, and 1% sodium deoxycholate with protease inhibitor cocktail [Roche, 4693132001]). The proteins with different molecular weights in cell lysates were separated by SDS‐PAGE and then transferred onto the PVDF membrane (Merck Millipore, IPVH00010) for immunoblot analysis. The following antibodies were used: anti‐GAPDH antibody (Proteintech, 60004‐1‐Ig), anti‐TOM20 antibody (Proteintech, 11802‐1‐AP), anti‐COXIV antibody (Proteintech, 11242‐1‐AP), anti‐HSP60 antibody (Proteintech, 66041‐1‐Ig), and anti‐p62 antibody (Santa Cruz, sc‐28359).

### 4.7 Immunofluorescence and Live Cell Imaging

The cells firstly were transfected with needed plasmids and then treated with indicated chemicals. Next, the cells were imaged using a live cell incubating system. Immunofluorescence assay was previously described (Wu et al. 2015; Yu et al. 2016; Chen et al. 2018; Ren et al. 2016; Fang et al. 2017). In brief, the cultured cells were fixed by paraformaldehyde (4%, 10 min) and then permeabilized by Triton X‐100 (0.1%, 10 min). Finally, the cells were stained by proper primary antibodies and matching fluorescent secondary antibodies. The following primary antibodies were used: anti‐TOM20 antibody (Proteintech, 11802‐1‐AP), anti‐p62 (Santa Cruz, sc‐28359), anti‐p62 (Cell signaling technology, D6M5X), anti‐p62 (Selleck, D16H4), anti‐HSP60 (Proteintech, 66041‐1‐Ig), anti‐Ub (Enzo Life Sciences, ENZ‐ABS840), anti‐HA antibody (Santa Cruz, sc‐7392), and anti‐MAP2 (Proteintech, 17490‐1‐AP). The cells were visualized using a Nikon fluorescent microscope (Ti2) with a pco.edge 4.2 bi sCMOS camera or a Nikon (A1R) confocal microscope with NSPARC super resolution imaging system. For in vitro imaging of p62 and mitochondria, the lysate was co‐incubated with primary and secondary antibodies (1:1000) at room temperature for 1 h.

### 4.8 Activation, Recovery After Activation, and Fluorescence Recovery After Photobleaching (FRAP) on Optogenetic Model

The optogenetic tool was activated by blue light (0.5%, 1% or maximum power, 488 nm wavelength) for 1 s or continuous stimulation in cells, and then the cells were imaged by 561 nm wavelength for us to observe the condensate recovery process. For FRAP, the condensates were photobleached by 40% power of 488 nm light (for green fluorescence) or 561 nm light (for red fluorescence) on showed space and then were imaged to show the fluorescence recovery process. In quantification of FRAP results, the intensity of pre‐photobleaching droplets was normalized to 100% and the first photobleaching droplets were normalized to 0%. In Opto‐p62‐mediated mitochondrial clustering experiment, p62‐depleted cells expressing Opto‐p62 were first pre‐treated by 5 μg/mL A/O for 1.5 h, and then were activated by blue light (488 nm wavelength) and imaged by 561 nm wavelength for us to observe the mitochondrial clustering process.

### 4.9 Statistical Analysis

Immunoblot densitometry was performed by Image processing and analysis in Java (ImageJ) software. Cellular localization of mitochondria, LC3, OPTN, p62, and Ub was determined by ImageJ software. Mitochondria track displacement, 3D reconstruction, morphometry, and area analyses were performed by Imaris 9.0.1. The charts about the obtained data were performed by Prism 8.0 (GraphPad) software. *p*‐values were displayed in figure legends. The formula of compaction index (CI; Narendra et al. 2010) indicates the circled perimeter with the same area as the interested object (*P*_0_) divided by the actual perimeter of the interested object (*P*_real_), and the following is the specific formula:

$$\text{Compaction Index}\;\left(\mathrm{CI}\right)=\frac{P_{0}}{P_{\text{real}}}=\frac{2\pi \sqrt{\frac{{\text{Area}}_{\text{real}}}{\pi }}}{P_{\text{real}}}$$

The relaxation time (*τ*; Ravindran et al. 2023; Gordon et al. 2025) was obtained using NonLinearRegression function of aspect ratio (A.R.) over time (*t*), and the following is the specific equation (A.R._0_ indicates initial aspect ratio):

$$A.R.=1+\left(A.R._{0}-1\right)\cdot \exp\left(-\frac{t}{\tau }\right)$$

## Author Contributions

S.S., J.X., K.Y.T., H.W., and Z.Y. conceptualization; S.S., J.X., Y.Z., B.Y., R.N., Q.M., H.W., and Z.Y. investigation; S.S., J.X., D.S., N.L., G.M., Q.P., S.C., K.Y.T., H.W., and Z.Y. formal analysis; S.S., J.H.M.P., K.Y.T., H.W., and Z.Y. writing and supervision.

## Funding

This study was supported by National Natural Science Foundation of China (Nos. 32471048, 32371018, 82022022, and 82071274), Jiangsu Key Laboratory of Drug Discovery and Translational Research for Brain Diseases (BM2013003), Natural Science Foundation of Jiangsu Provincial Higher Education Institutions (23KJA310005), Interdisciplinary Basic Frontier Innovation Program of Suzhou Medical College of Soochow University (YXY2301006), Suzhou International Joint Laboratory for Diagnosis and Treatment of Brain Diseases, Priority Academic Program Development of the Jiangsu Higher Education Institutes (PAPD), the Science and Technology Development Fund, Macau SAR (File no. 0062/2021/A), and the University of Macau (File no. MYRG2022‐00171‐FHS).

## Conflicts of Interest

The authors declare no conflicts of interest.

## Supporting information


**Figure S1:** The liquid‐like property of p62 condensates.
**Figure S2:** ALS/FTD‐associated p62 mutations alter the property of p62 condensates.
**Figure S3:** p62 deficiency accelerates mitochondrial clearance and ALS/FTD‐associated p62 mutations influence mitochondrial clustering.
**Figure S4:** ALS/FTD‐associated p62 mutations influence mitochondrial clearance.

**Video S1:** Blue light photoactivation and recovery of Cry2‐mCherry (as a control) and Opto‐p62 in live cells.

**Video S2:** Repeated photoactivation and recovery of WT and mutant Opto‐p62 condensates in live cells.

## Data Availability

The data that support the findings of this study are available on request from the corresponding author. The data are not publicly available due to privacy or ethical restrictions.
