# Supplementary material for: p62/SQSTM1 Condensation Modulates Mitochondrial Clustering to Participate in Mitochondrial Quality Control
Source: Aging Cell. 2026 Feb 11;25(2):e70402. doi: 10.1111/acel.70402 (PMC12894778; doi:10.1111/acel.70402)
Supplement: Supplementary file 1 — Figure S1: The liquid‐like property of p62 condensates. Figure S2: ALS/FTD‐associated p62 mutations alter the property of p62 condensates. Figure S3: p62 deficiency accelerates mitochondrial clearance and ALS/FTD‐associated p62 mutations influence mitochondrial clustering. Figure S4: ALS/FTD‐associated p62 mutations influence mitochondrial clearance. [file ACEL-25-e70402-s002.docx]

**Supplemental Figure 1**


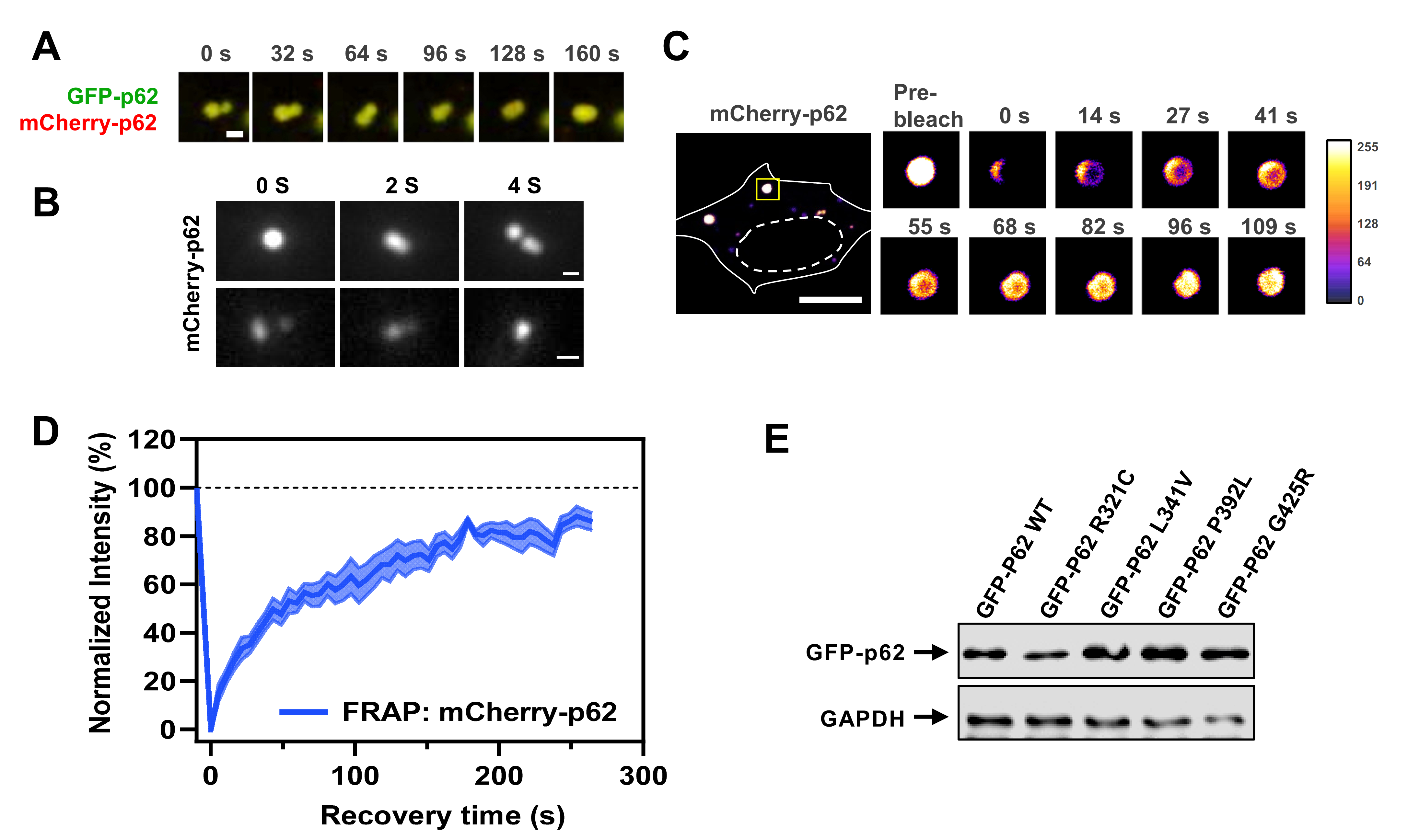


**Figure S1. The liquid-like property of p62 condensates.** (**A**) HEK 293 cells were transfected with GFP-p62 and mCherry-p62 for 24 h, and then were imaged using a live cell imaging system. Scale bar, 1 μm. (**B**) Fusion and division of p62 bodies formed by purified mCherry-p62 expressed in *Ecoli in vitro*. Scale bar, 2 μm. **(C)** HEK 293 cells were transfected with mCherry-p62 for 24 h, and then were subjected to FRAP analysis, representative images of mCherry-p62 puncta were shown. Scale bar, 10 μm. (**D**) Quantification of fluorescence intensity of mCherry-p62 cellular puncta in (**C**). Quantification data are shown as mean ± SEM from three independent experiments. (**E**) p62-deficient HEK 293 cells were transfected with GFP-p62 WT, R321C, L341V, P392L, or G425R for 24 h, and then the cell lysates were collected and subjected to immunoblot with p62 and GAPDH antibodies.

**Supplemental Figure 2**


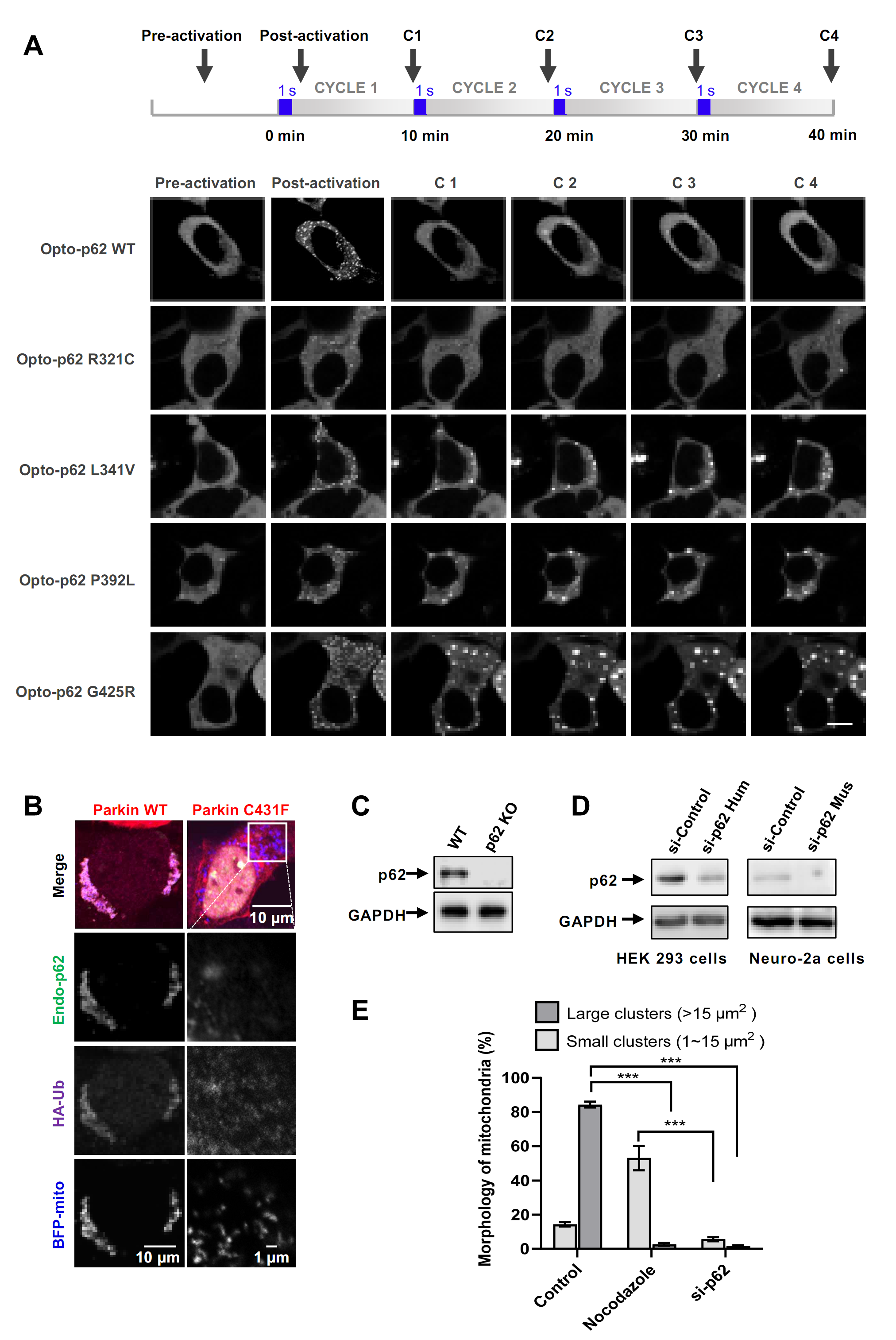


**Figure S2. ALS/FTD-associated p62 mutations alter the property of p62 condensates.** (**A**) HEK 293 cells were transfected with Opto-p62 (WT, R321C, L341V, P392L, or G425R), and then activated by four cycles of blue light stimulation. Scale bar, 10 μm. See also Video S2. (**B**) HEK 293 cells were transfected with mCherry-Parkin (WT or C431F) and BFP-mito for 24 h, and then were treated with 5 μg/ml Antimycin/Oligomycin (A/O) for 4 h. The cells were subjected to immunofluorescent assay using anti-p62 and anti-Ub antibodies. Scale bar, 1 μm or 10 μm. (**C**) Immunoblots analysis showed knockout efficiency of p62 from CRISPR-Cas9-generated U-2 OS cells. (**D**) Left: HEK 293 cells were transfected with control (non-targeting oligonucleotide, si-Control) or siRNA targeting human p62 (si-p62 Hum) for 48 h; Right: Neuro-2a cells were transfected with si-Control or siRNA targeting mouse p62 (si-p62 Mus) for 48 h. Then, the cell lysates were collected and subjected to immunoblot with indicated antibodies. (**E**) Quantification of mitochondrial clusters in Figure 4B. Quantification data are shown as mean ± SEM from three independent experiments. **^***^***p*<0.001, one-way Anova followed by post hoc Tukey’s tests.

**Supplemental Figure 3**


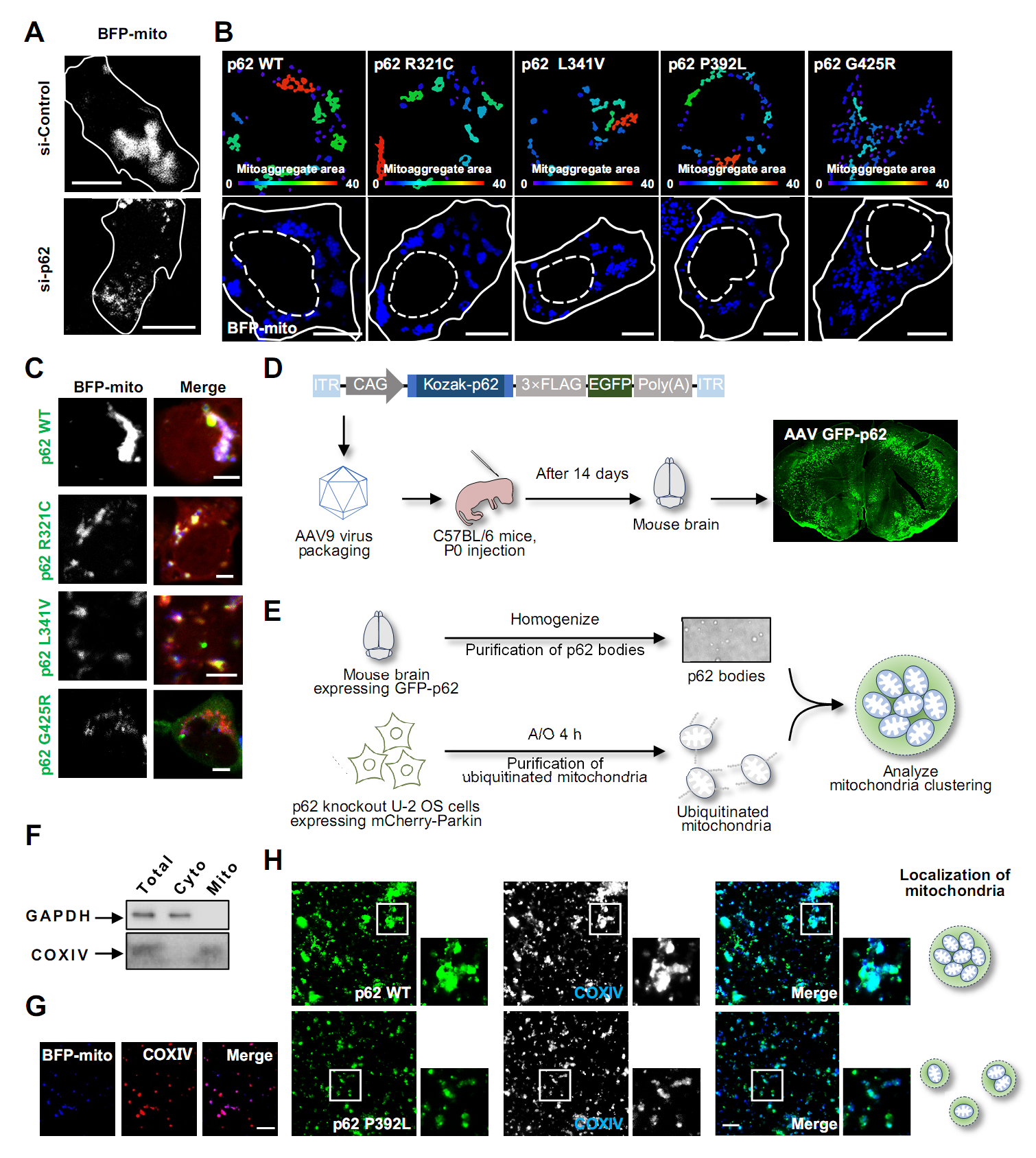


**Figure S3. p62 deficiency accelerates** **mitochondrial clearance and** **ALS/FTD-associated p62 mutations influence mitochondrial** **clustering.** (**A**) SH-SY5Y cells were transfected with si-Control or siRNA targeting human p62, and then were re-transfected with mCherry-Parkin and BFP-mito. The cells were treated with 5 μg/ml A/O for 4 h. Scale bar, 10 μm. (**B**) p62-deficient HEK 293 cells were re-transfected with mCherry-p62 (WT, R321C, L341V, P392L or G425R), HA-Parkin and BFP-mito, and then were treated with 5 μg/ml A/O treatment for 1.5 h. Scale bar, 10 μm. Upper panels: 3D reconstruction of mitochondrial cluster volumes. Warmer colors in the color bar indicate increasing mitochondrial clustering. (**C**) p62-deficient SH-SY5Y cells were re-transfected with GFP-p62 (WT, R321C, L341V or G425R), mCherry-Parkin and BFP-mito, and then were treated with 5 μg/ml A/O treatment for 4 h. Scale bar, 5 μm. (**D**) The C57BL/6 neonatal mice (P0) were intra-cerebroventricular injection (ICV) injected by AAV-GFP-p62 (WT or P392L) for 14 days, and then were sacrificed and the brains were used by frozen sectioning and imaging. (**E**) Schematic of mitochondrial clustering mediated by purified p62 bodies from mouse brain homogenates. (**F**) p62-deficient HEK 293 cells were transfected with GFP-Parkin and BFP-mito. After treatment with or without 5 μg/ml A/O for 2 h, cell lysates were collected and subjected to mitochondrial isolation and immunoblot analysis with indicated antibodies. The experiment was repeated for three independent times. (**G**) COXⅣ antibody combined with secondary antibody conjugated to Alexa 594 was used to verify that purified efficiency of mitochondria purification. Scale bar, 10 μm. (**H**) Representative images of mitochondrial clustering mediated by p62 bodies. The purified mitochondria combined with p62 bodies, COXⅣ antibody and secondary antibody conjugated to Alexa 405 in buffer (150 mM NaCl, 50 mM Tris-HCl, 2 mM DTT, 5% PEG4000) for 30 min. Scale bar, 20 μm.

**Supplemental Figure 4**


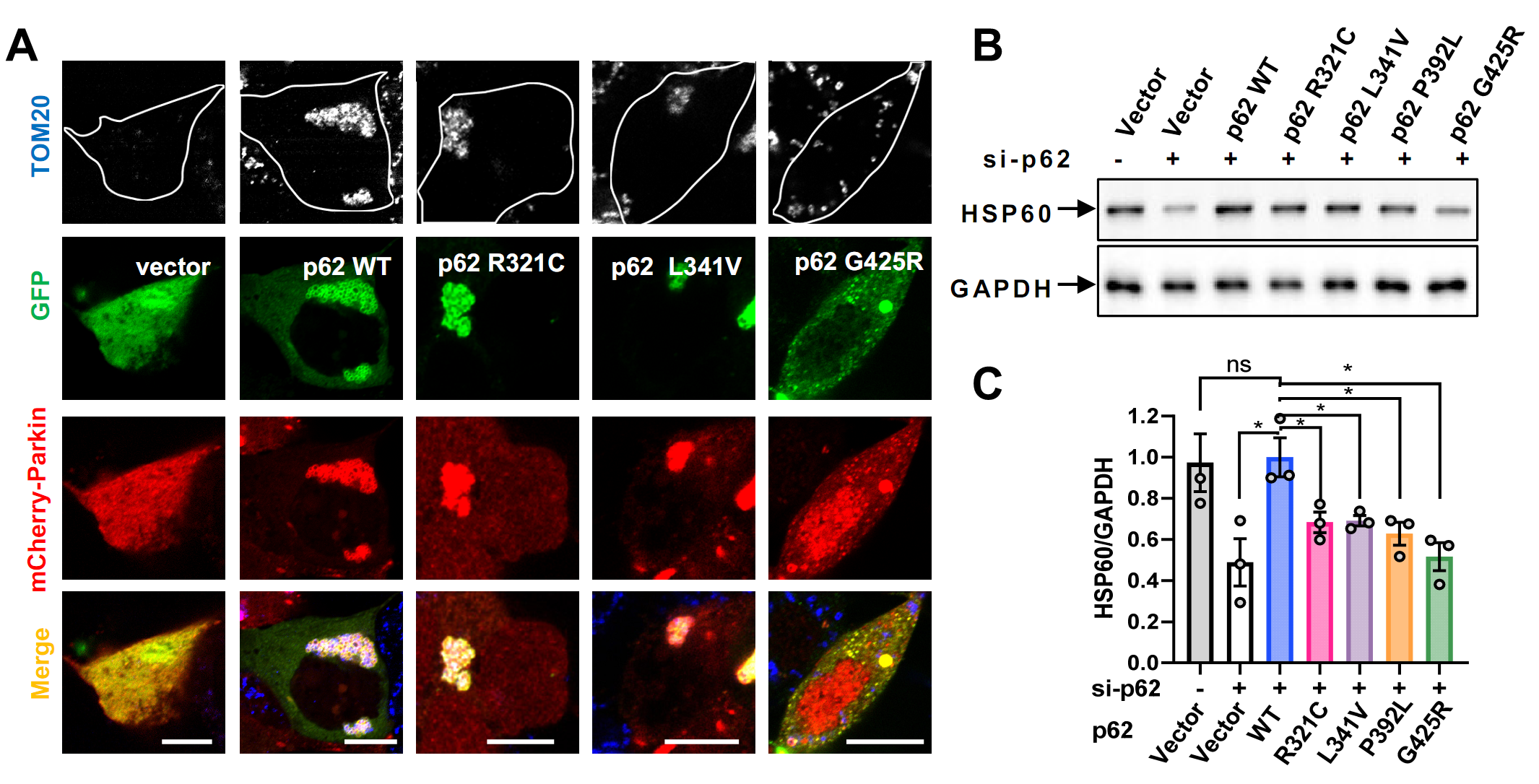


**Figure S4. ALS/FTD-associated p62 mutations influence mitochondrial clearance.** (**A**) HEK 293 cells were transfected with p62 siRNA for 24 h, and then were re-transfected with mCherry-Parkin as well as GFP-vector or GFP-p62 (WT, R321C, L341V or G425R) for 24 h. After treatment with 1 μg/ml A/O for 16 h, the cells were stained with TOM20 antibody using Alexa 647 nm fluorescence secondary antibody. Scale bar, 10 μm. (**B**) HEK 293T cells were transfected with control or p62 siRNA for 24 h, and then were re-transfected with FLAG-Parkin and GFP-vector or GFP-p62 (WT, R321C, L341V, P392L or G425R) for 24 h. After 1 μg/ml A/O treatment for 16 h, cells were analyzed by immunoblot with the indicated antibodies. (**C**) Quantification of the relative protein levels in (**B**). Data are shown as mean ± SEM from three independent experiments. **^*^***p*<0.05; ns, not significant, t-test.
